# Leaf hydraulic conductance is coordinated with leaf morpho-anatomical traits and nitrogen status in the genus *Oryza*

**DOI:** 10.1093/jxb/eru434

**Published:** 2014-11-26

**Authors:** Dongliang Xiong, Tingting Yu, Tong Zhang, Yong Li, Shaobing Peng, Jianliang Huang

**Affiliations:** National Key Laboratory of Crop Genetic Improvement, MOA Key Laboratory of Crop Ecophysiology and Farming System in the Middle Reaches of the Yangtze River, College of Plant Science and Technology, Huazhong Agricultural University, Wuhan, Hubei 430070, China

**Keywords:** CO_2_ diffusion conductance, leaf anatomy, leaf hydraulic conductance, leaf N content per leaf area, photosynthesis, rice.

## Abstract

Leaf hydraulic conductance (*K*_leaf_) is a major determinant of photosynthetic rate in plants. Previous work has assessed the relationships between leaf morpho-anatomical traits and *K*_leaf_ with woody species, but there has been very little focus on cereal crops. The genus *Oryza*, which includes rice (*Oryza sativa*) and wild species (such as *O. rufipogon* cv. Griff), is ideal material for identifying leaf features associated with *K*_leaf_ and gas exchange. Leaf morpho-anatomical traits, *K*_leaf_, leaf N content per leaf area, and CO_2_ diffusion efficiency were investigated in 11 *Oryza* cultivars. *K*_leaf_ was positively correlated with leaf thickness and related traits, and therefore positively correlated with leaf mass per area and leaf N content per leaf area, and negatively with inter-veinal distance. *K*_leaf_ was also positively correlated with leaf area and its related traits, and therefore negatively correlated with the proportion of minor vein length per area. In addition, coordination between *K*_leaf_ and CO_2_ diffusion conductance in leaves was observed. We conclude that leaf morpho-anatomical traits and N content per leaf area strongly influence *K*_leaf_. Our results suggest that more detailed anatomical and structural studies are needed to elucidate the impacts of leaf feature traits on *K*_leaf_ and gas exchange in grasses.

## Highlight

The range of leaf hydraulic conductance across the genus *Oryza* is caused by leaf morpho-anatomical traits and leaf N status.

## Introduction

Leaf hydraulics is the major bottleneck of the overall plant hydraulic system, and therefore the fundamental factor restricting gas exchange and biomass production (Sack *et al.*, 2003; Sack and Holbrook, 2006). The efficiency of water transport through the leaf to the evaporating surface of the mesophyll is quantified by leaf hydraulic conductance (*K*_leaf_), which is generally expressed on a leaf area base (Sack and Holbrook, 2006). Decreases in *K*_leaf_ usually cause leaves to become less hydrated (corresponding to a low leaf water potential), a response often associated with stomatal closure and, consequently, reduced CO_2_ assimilation (Sperry, 2000; Johnson *et al.*, 2009). This reduction occurs partly because CO_2_ and water exchange between leaves and air share a common pathway through stomatal pores. The coupling of stomatal conductance (*g*_s_) to CO_2_ and water vapour leads to strong coordination between *g*_s_ and *K*_leaf_ (Sack *et al.*, 2003; Brodribb *et al.*, 2005; Sack and Holbrook, 2006). Furthermore, owing to the tight coupling between *g*_s_ and photosynthetic rate (*A*) in C_3_ species (Wong *et al.*, 1979), a positive relationship between *K*_leaf_ and *A* is reported (Brodribb *et al.*, 2005; Franks, 2006; Brodribb *et al.*, 2007; Flexas *et al.*, 2013*b*).

Previous studies have found that *K*_leaf_ varies greatly between species, ranging 65-fold from the lowest to highest value (Sack and Holbrook, 2006). Interspecific variation in *K*_leaf_ reflects differences in the morpho-anatomy of leaves, as well as pathways through the outside xylem to evaporation sites. In plants, leaf vein systems, as distinct water transport systems, vary greatly in arrangement, density, vascular bundle features, and xylem conduits within the bundles (Sack and Scoffoni, 2013). In the past two decades, increasing numbers of studies have focused on the relationship between *K*_leaf_ and venation architecture, expressed as vein length per area (VLA). Positive and negative relationships between *K*_leaf_ and VLA have been reported (Nardini *et al.*, 2012; Sack and Scoffoni, 2012;), although no relationship was found in another study (Flexas *et al.*, 2013*b*). Most of these studies, however, were conducted with woody species, and very few focused on cereal crops such as rice.

Outside the xylem, there are three main pathways for water flow: apoplastic, symplastic, and transcellular (Sack and Holbrook, 2006). Several early studies suggested that water exits the xylem mainly through the apoplastic pathway, because of high resistance in the symplastic and transcellular pathways. However, many recent studies have shown that aquaporins have a positive effect on water transport across the membranes of bundle sheath and mesophyll cells (Martre *et al.*, 2002; Sack *et al.*, 2004). Dye and cell pressure probe experiments also suggest that the symplastic and transcellular pathways play a vital role in water transport in plants (Murphy and Smith, 1998; North *et al.*, 2013). Furthermore, the distance that water travels from veins to stomata (*D*_s_), which is usually expressed as the distance between veins and stomata (*D*_m_) in the leaf cross section (Brodribb *et al.*, 2007; North *et al.*, 2013), has been suggested to be an important trait affecting *K*_leaf_. Although water movement in mesophyll tissues is now widely recognized, how leaf mesophyll architecture contributes to water flux in the mesophyll and water evaporation at the cell wall surface remains unclear (Sack and Holbrook, 2006; Flexas *et al.*, 2013*b*; North *et al.*, 2013).

N is a vitally important element for plants, and it profoundly influences leaf anatomical and functional traits (Rademacher and Nelson, 2001; Lee *et al.*, 2011). Previous studies have shown that leaf N promotes *A* by increasing Rubisco content and CO_2_ diffusion conductance (Imai *et al.*, 2008; Franks *et al.*, 2009). However, the correlation of leaf N content per leaf area with *K*_leaf_ remains to be investigated. Studying the interactions between leaf N status and *K*_leaf_ may help determine the effects of N on rice leaf morpho-anatomical traits associated with *K*_leaf_ and CO_2_ movement in leaves.

*Oryza* spp. are distributed worldwide, and they exhibit a wide range of phenotypes. This diversity is an important resource (Giuliani *et al.*, 2013) that is being utilized to improve rice yield and other agronomic traits, particularly in unfavourable environments. In the present study, four cultivated and seven wild cultivars in the genus *Oryza* were investigated with the aims of: (i) identifying the variation in leaf morpho-anatomical traits and *K*_leaf_; (ii) investigating whether leaf morpho-anatomical traits and leaf N status influence *K*_leaf_; and (iii) determining the relationship between *K*_leaf_ and gas exchange.

## Materials and methods

### Plant materials

Four rice (*O. sativa* L.) cultivars and seven wild cultivars in the genus *Oryza* (Table 1; these were provided by the National Key Laboratory of Crop Genetic Improvement) were investigated in a pot experiment at Huazhong Agricultural University, Wuhan, China. Three hills of seedlings were grown in 15.0 l pots filled with 13.0kg soil. N, P, and K were applied as basal fertilizers at a rate of 3.0g, 1.95g and 1.95g per pot, respectively. There were three pots per cultivar. Throughout their growth, plants were well watered and a water depth of at least 2cm was maintained. Pests were controlled using chemical pesticides.

**Table 1.** Leaf anatomical traits^a^

Cultivar	Species	Area (cm^2^)	Length (cm)	Width (cm)	VLA_major_ (mm mm^–2^)	VLA_minor_ (mm mm^–2^)	VLA (mm mm^–2^)	LMA (g m^–2^)	*K*_leaf_ (mmol m^–2^ s^–1^ MPa^–1^)
Shanyou 63	*O. sativa*	54.0±6.2	46.2±3.0	1.24±0.08	0.90±0.04	3.02±0.21	3.91±0.25	36.1±3.4	7.20±0.29
Huanghuazhan	*O. sativa*	31.7±1.4	28.0±2.1	1.36±0.15	0.74±0.08	2.81±0.39	3.56±0.47	43.3±1.5	8.74±0.73
N22	*O. sativa*	42.7±4.7	44.5±2.3	1.13±0.07	1.02±0.11	4.13±0.50	5.16±0.61	33.5±1.2	7.30±0.59
Nipponbare	*O. sativa*	31.2±2.4	25.1±2.2	1.40±0.10	0.71±0.02	2.27±0.04	2.98±0.16	40.8±3.4	7.17±1.19
Lat	*O. latifolia*	127.3±4.2	72.8±5.0	2.20±0.10	0.56±0.01	2.95±0.15	3.50±0.15	45.3±1.5	12.2±0.4
Aus	*O. australiansis*	43.7±8.0	40.4±1.5	1.31±0.09	0.98±0.06	2.97±0.21	3.95±0.27	38.6±1.5	4.93±0.89
I08	*O. rufipogon*	31.0±1.1	41.0±3.2	0.80±0.09	1.03±0.08	3.42±0.26	4.45±0.34	26.9±1.0	3.63±0.55
I90	*Oryza. punctata*	38.4±0.7	50.1±3.0	1.00±0.11	1.08±0.03	3.83±0.12	4.90±0.15	39.9±3.9	5.76±0.21
Wcr	*Oryza. granulata*	21.0±3.2	27.4±1.9	0.38±0.06	1.88±0.07	4.58±0.37	6.46±0.44	33.7±0.8	4.30±0.94
Ruf	*Oryza. rufipogon*	30.5±3.5	24.1±2.7	1.19±0.06	1.04±0.03	3.40±0.17	4.44±0.19	33.4±1.6	4.09±0.31
Rhi	*Oryza. rufipogon*	18.4±1.8	16.0±1.0	1.45±0.11	0.67±0.02	2.14±0.11	2.81±0.12	24.6±1.1	3.31±0.23
Analysis of variance									
Average		43.1±29.9	37.8±16.0	1.21±0.44	0.97±0.35	3.23±0.74	4.20±1.10	36.0±3.5	6.24±2.65
Cultivars		***	***	***	***	***	***	***	***

^a^ Values are mean ± SD; ***, *P* < 0.001.

### Gas exchange measurements

To avoid the effect of fluctuation in outdoor air temperature, light intensity, and humidity on gas exchange measurement, measurement was done between 9.30 and 15.30 in an environmentally controlled room with an air temperature of 27.8±2.1°C, a photosynthetic photon flux density (PPFD) at the leaf surface of 1200±47 μmol m^–2^ s^–1^(artificial light source), and relative humidity of 77.4±5.3%. Measurements were taken on newly and fully expanded leaves of three plants for each cultivar after they were acclimated for ~1.5h. Gas exchange and chlorophyll fluorescence were simultaneously measured using an LI-6400XT portable photosynthesis system equipped with a leaf chamber (LI-COR, NE, USA). Leaf temperature during measurements was maintained at 28°C. In the leaf chamber, PPFD was maintained at 1500 μmol m^–2^ s^–1^, and leaf-to-air vapour pressure deficit at 1.1–1.4 kPa; CO_2_ concentration was adjusted to 400 μmol m^–2^ s^–1^ with a CO_2_ mixture. After equilibration to a steady state, *A*, *g*_s_, steady-state fluorescence (*F*_s_), and maximum fluorescence (*F*_m_ʹ) were recorded. The actual photochemical efficiency of photosystem II (*Φ*_PSII_) was calculated as follows:

$$\Phi_{\;PSII}\text{=}\frac{\left(F_{\text{m}}^{\prime }-F_{\text{s}}\right)}{F_{\text{m}}^{\prime }}$$

Electron transport rate (*J*) was calculated as follows:

$$J=\Phi_{\text{ PSII}}\cdot \text{PPFD}\cdot \alpha \beta$$

where *α* is the leaf absorptance and *β* represents the distribution of electrons between PSI and PSII.

Light response curves were determined under low O_2_ concentration (<2%) for estimating *α* and *β*. The gas exchange system was immediately switched to low O_2_ concentration (<2%) without removing the leaves from the chamber. Simultaneous measurements of light response curves and chlorophyll fluorescence were then performed. During the measurements, chamber conditions were the same as those described above, except that PPFD was controlled across a series: 2000, 1200, 800, 400, 250, 200, 150, 100, 50, 20, and 0 µmol m^–2^ s^–1^. After reaching a steady state, parameters of gas exchange and chlorophyll fluorescence were simultaneously recorded. The values of *αβ* and daytime respiration rate (*R*_d_) were calculated as the slope and intercept, respectively, of the linear regression of *A* on PPFD·*Φ*_PSII_/4 (Yin *et al.*, 2009). Our *αβ* values are consistent with the values estimated from the slope between *Φ*_PSII_ and *Φ*_CO2_ with varying light intensity under non-photorespiratory conditions (O_2_ < 1%) (Supplementary Figure S1).

The variable *J* method described in Harley *et al.* (1992) was used to calculate mesophyll conductance of CO_2_ (*g*_m_) and CO_2_ concentration in the chloroplast (*C*_c_). *C*_c_ was calculated as follows:

$$C_{\text{c}}=\frac{\Gamma *\left(J+\text{8}\left(A+R_{\text{d}}\right)\right)}{J-\text{4}\left(A+R_{\text{d}}\right)}$$

where Γ* represents the CO_2_ compensation point in the absence of respiration. Г* is related to Rubisco-specific factor, which is relatively conserved under given temperature conditions (Bernacchi *et al.*, 2002; Warren and Dreyer, 2006). In the present study, a Г* value of 40 μmol mol^–1^, typical for *Oryza* plants, was taken based on the studies of Franks *et al.* (2009) and Giuliani *et al.* (2013). Then, *g*_m_ was calculated as follows:

$$g_{\text{m}}=\frac{A}{C_{\text{i}}-C_{\text{c}}}$$

where *C*_i_ represents the intercellular CO_2_ concentration.

### Leaf hydraulic conductance

*K*_leaf_ was measured using the evaporative flux method (Sack *et al.*, 2002; Brodribb *et al.*, 2007; Guyot *et al.*, 2012; Sack and Scoffoni, 2012). Three to nine leaves of each cultivar were excised in water and placed under conditions favourable to transpiration (i.e. PPFD of 1200 µmol m^−2^ s^−1^ and air temperature of 28°C) with the petiole attached to a potometer. When leaves reached a transpirational steady state, the transpirational flux rate (*E*) was recorded. The leaf area was then measured using a leaf area meter (LI-Cor 3000C, LI-COR, NE, USA) and leaf length and width were measured quickly using a plastic ruler. The leaves were detached and cut into small sections, immediately followed by leaf water potential (*Ψ*_leaf_) measurement using a WP4C Dewpoint PotentiaMeter (Decagon, Pullman, WA, USA). *K*_leaf_ was calculated as follows:

$$K_{\text{leaf}}\text{=}\frac{E}{0-\Psi_{\text{leaf}}}$$

### VLA and leaf thickness

Three leaves per cultivar were cleared in 20% aqueous NaOH after their widths were recorded. Three sections of leaf lamina of ~5.0mm length were excised from the middle portion of each leaf, stained, and mounted in glycerol for the determination of vein density. According to Scarpella *et al.* (2003) and Smillie *et al.* (2012), rice vascular bundles can be categorized into three types based on their size: midrib, large veins, and minor veins. In the present study, the numbers of major veins (sum of midrib and large veins) and minor veins, and the inter-vein distance (IVD, distance between two minor veins), were recorded using a microscope at 40× magnification. The proportion of minor vein length was calculated as the percentage of minor vein length per area (VLA_minor_) over VLA. The leaf thickness (*T*_leaf_) was measured using a DTG03 digital thickness gauge (Digital Micrometers Ltd, Sheffield, UK).

### Leaf N content per leaf area

After *Ψ*_leaf_ measurement, leaves were oven-dried at 80°C to constant weight, and ground using a mixer mill homogenizer (MM400, Retsch, Germany). Approximately 5.0mg was used to measure N content per leaf area using an NC analyzer (IsoPrime100 IRMS, Isoprime Ltd, UK).

### Statistical analyses

One-way analysis of variance (ANOVA) and multiple regression analysis were applied to assess the significance of cultivar effect with SAS 9.2 (SAS Institute Inc., USA). Regression analyses between parameters were performed using SigmaPlot 12 (SPSS Inc., Chicago, IL, USA). All regressions were fitted by both linear and power models, and the model with higher regression coefficient was selected.

## Results

### Differences in leaf morpho-anatomical traits and *K*_leaf_ across cultivars

There were very large variations in leaf morpho-anatomical traits in the genus *Oryza* (Table 1). The differences were 6.9-fold in leaf area (ranging from a minimum of 18.4cm^2^ in Rhi to a maximum of 127.3cm^2^ in Lat), 4.6-fold in leaf length, and 5.8-fold in leaf width. With respect to leaf veins, VLA, VLA_major_, and VLA_minor_ were significantly different across cultivars. There was a 2.3-fold difference in VLA (minimum in Rhi and maximum in Wcr). The difference in leaf mass per area (LMA) was 1.8-fold (minimum in Rhi and maximum in Lat), and the difference in *K*_leaf_ was 3.7-fold (minimum in Rhi and maximum in Lat).

### Relationships among leaf morpho-anatomic traits, leaf N, and *K*_leaf_

**Fig. 1.**
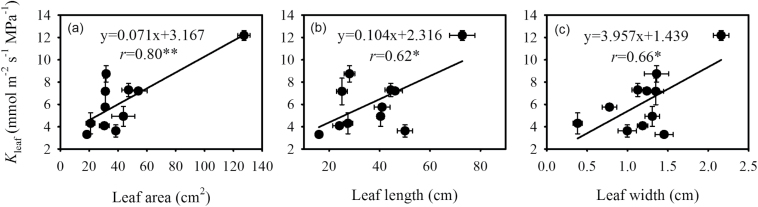
Relationships between leaf hydraulic conductance (*K*_leaf_) and (A) leaf area, (B) leaf length, and (C) leaf width. The values shown are mean ± SD, and data were fitted by linear regression. Regression coefficients and significance are shown when *P* < 0.05 (*, *P* < 0.05; **, *P* < 0.01).

**Fig. 2.**
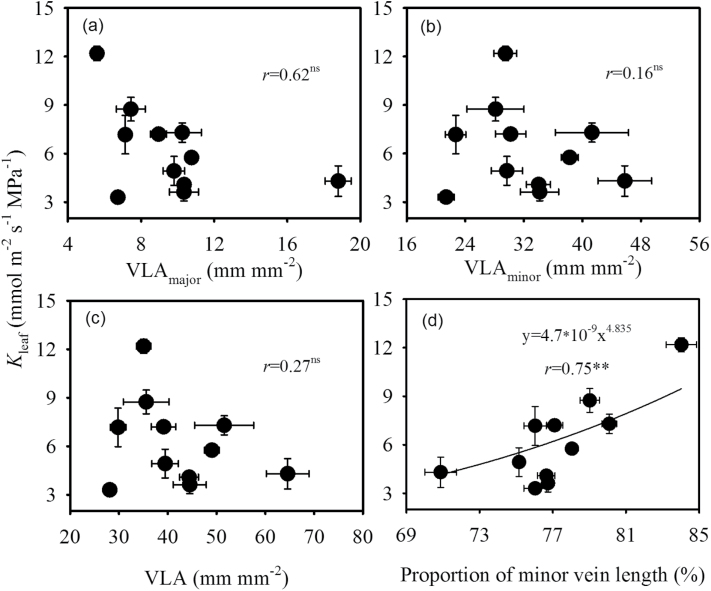
Relationships between leaf hydraulic conductance (*K*_leaf_) and (A) VLA_major_, (B) VLA_minor_, (C) VLA, and (D) proportion of minor vein length. Values shown are mean ± SD, and data in (D) were fitted by power regression. Regression coefficients and significance are shown when *P* < 0.05 (**, *P* < 0.01; ns, not significant).

**Fig. 3.**
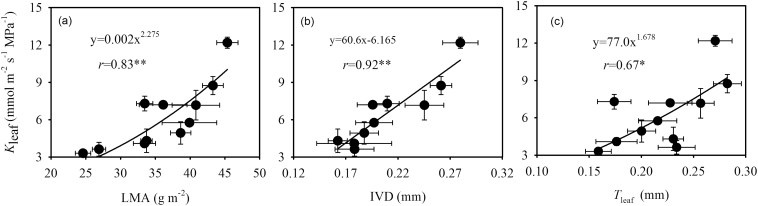
Relationship between *K*_leaf_ and (A) LMA, (B) IVD, and (C) *T*_leaf_. Values shown are mean ± SD, and data were fitted by power adjustment. Regression coefficients and significance are shown when *P* < 0.05 (*, *P* < 0.05; **, *P* < 0.01).

**Fig. 4.**
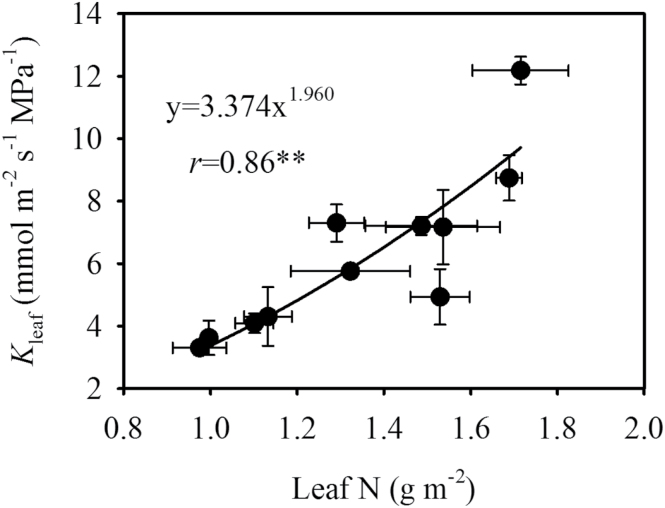
Correlation of leaf N content per leaf area and *K*_leaf_. Values shown are mean ± SD, and data were fitted by power adjustment. Regression coefficients and significance are shown when *P* was <0.05 (**, *P* < 0.01).

**Fig. 5.**
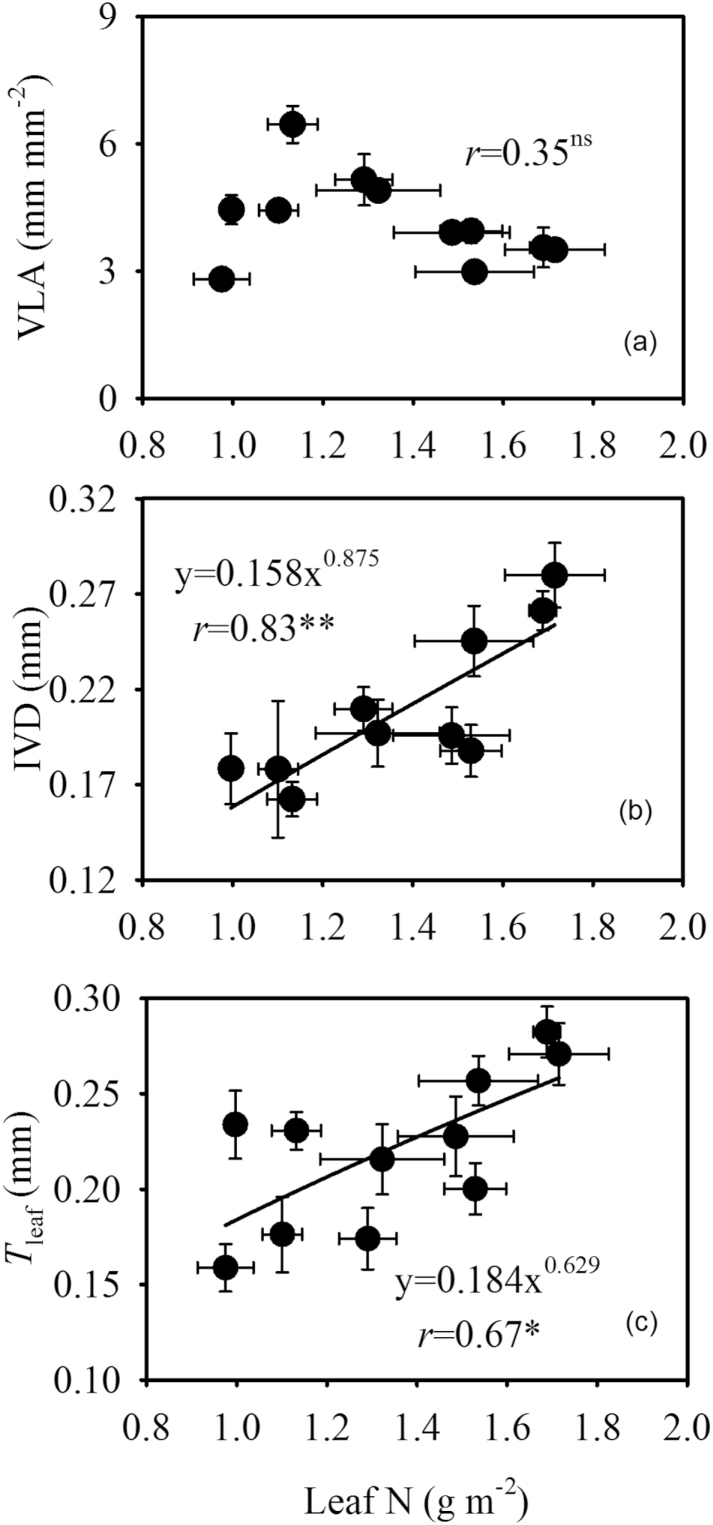
Effect of leaf N content per leaf area on (A) VLA, (B) IVD, and (C) *T*_leaf_. Values shown are mean ± SD, and data in (B) and (C) were fitted by power adjustment. Regression coefficients and significance are shown when *P* < 0.05 (ns, not significant; *, *P* < 0.05; **, *P* < 0.01).

Across all cultivars, *K*_leaf_ was positively correlated with leaf area (*r* = 0.80, *P* < 0.01), leaf length (*r* = 0.62, *P* < 0.05), and leaf width (*r* = 0.66, *P* < 0.05) (Fig. 1). No significant correlation was observed between *K*_leaf_ and VLA, VLA_major_, or VLA_minor_. However, a positive correlation (*r* = 0.86, *P* < 0.01) between the proportion of minor vein length and *K*_leaf_ was observed (Fig. 2). In addition, *K*_leaf_ was positively correlated with LMA (*r* = 0.83, *P* < 0.01), IVD (*r* = 0.92, *P* < 0.01), *T*_leaf_ (*r* = 0.67, *P* < 0.05) (Fig. 3), and leaf N content per leaf area (*r* = 0.86, *P* < 0.01) (Fig. 4). IVD and *T*_leaf_ were positively correlated with leaf N content per leaf area, while VLA was independent of leaf N content per leaf area (Fig. 5). In order to identify the direct effects of leaf N content per leaf area on *K*_leaf_, a multiple regression analysis was performed between *K*_leaf_ and leaf N content per leaf area, *T*_leaf_, and IVD. Our results show that *K*_leaf_ tightly correlated with N content per leaf area (*P* = 0.015) compared with *T*_leaf_ (*P* = 0.673) and IVD (*P* = 0.052).

### Relationship between *K*_leaf_ and gas exchange

There were very large variations in *A*, *g*_s_, and *g*_m_ in the genus *Oryza* (Supplementary Figure S2). The *g*_m_ estimated by a combination of gas-exchange and chlorophyll fluorescence methods showed a linear relationship with the value estimated from the *A-C*_*i*_ curve-fitting method (Supplementary Figure S1). Across all cultivars, a positive correlation (*r* = 0.63, *P* < 0.05) was found between *A* and *K*_leaf_ (Table 2; Supplementary Figure S2). *A* was closely related to total CO_2_ diffusion conductance (*g*_t_) (*r* = 0.85, *P* < 0.01), *g*_s_ (*r* = 0.86, *P* < 0.01) and *g*_m_ (*r* = 0.73, *P* < 0.01). The *g*_t_ was positively correlated with both *g*_s_ (*r* = 0.92, *P* < 0.001) and *g*_m_ (*r* = 0.92, *P* < 0.001). There was a strong relationship between *g*_s_ and *g*_m_. *K*_leaf_ was positively correlated with *g*_t_ (*r* = 0.88, *P* < 0.01), *g*_s_ (*r* = 0.75, *P* < 0.01), and *g*_m_ (*r* = 0.77, *P* < 0.01).

**Table 2.** Coefficients of correlations^a^

	***K***_**leaf**_	***A***	***g***_**t**_	***g***_**s**_	***g***_**m**_
***K***_**leaf**_	1.00***	0.63*	0.88**	0.75**	0.77**
***A***		1.00***	0.85**	0.86**	0.73**
***g***_**t**_			1.00***	0.92***	0.94***
***g***_**s**_				1.00***	0.73**
***g***_**m**_					1.00***

^a^ *, *P* < 0.05; **, *P* < 0.01; ***, *P* < 0.001.

## Discussion

### Relationship between *A* and *K*_leaf_

Improving photosynthesis is central to improving crop yield. In C_3_ plants, an important determinant of photosynthesis is the CO_2_ concentration in the chloroplast. (Evans and Von Caemmerer, 1996; Flexas *et al.*, 2008; Franks *et al.*, 2009; Flexas *et al.*, 2013*a*). Previous studies have shown correlations between *A* and *K*_leaf_ across a wide range of species (Brodribb *et al.*, 2007; Flexas *et al.*, 2013*b*). In the present study, *A* was correlated with *K*_leaf_ in the genus *Oryza* (Table 2). During photosynthesis, CO_2_ must move from outside the leaf through the stoma to the sub-stomatal internal cavities, and from there to the site of carboxylation inside the chloroplast though leaf mesophyll (Evans *et al.*, 2009; von Caemmerer and Evans, 2010; Flexas *et al.*, 2012). Opening the stomata would benefit photosynthesis in the presence of sufficiently high intercellular CO_2_ concentration. However, maintaining open stomata depends on leaf water supply capacity, which is determined by *K*_leaf_. Under normal conditions, *K*_leaf_ is limited by leaf anatomy (Sack *et al.*, 2003; Sack and Holbrook, 2006).

### Relationship between *K*_leaf_ and leaf morpho-anatomical traits

Across a large variation in leaf area, we observed a positive correlation between *K*_leaf_ and leaf area (Fig. 1), as was also observed in *Acer* and *Quercus* spp. (Nardini *et al.*, 2012). However, our results were contrary to those of Simonin *et al.* (2012), who showed, by summarizing published data, that *K*_leaf_ was independent of variations in leaf area. There are two reasons for the discrepancy between our results and those of Simonin *et al.* (2012). Firstly, our results were derived from the genus *Oryza*, which has a homologous hydraulic architecture, and the relatively expanded (leaf area and leaf thickness increasing) leaf needs to evolve stronger water transportation ability, because vein xylem conductivity tends to increase with leaf size. However, the result reported by Simonin *et al.* (2012) was derived from a wide range of plant species with a multiplicity of leaf hydraulic architectures, masking the effects of leaf area and leaf thickness on *K*_leaf_. Secondly, the large variation in leaf area in the present study was contributed by Lat (Table 1; Fig. 1), which caused a significant correlation between *K*_leaf_ and leaf area.

In the present study, a strong positive correlation was observed between *K*_leaf_ and LMA (Fig. 3). If LMA is considered as the sum of the mass of different leaf tissues per unit of leaf area, variation in LMA occurs via changes in leaf tissue composition. Blonder *et al.* (2011), on the basis of a mathematic model, hypothesized that high VLA results in high LMA. However, Sack *et al.* (2013) contested this by compiling a large database, reporting that, in fact, vein xylem and sclerenchyma accounted for <10% of leaf volume per area and thus did not contribute strongly and directly to either leaf thickness or leaf density (Sack *et al.*, 2013). In the present study with the genus *Oryza*, no relationship between VLA and LMA was observed (Supplementary Figure S3). Additionally, especially within species, LMA correlates with *T*_leaf_, which is derived from layers of mesophyll cells. Our result indicates that the variation in LMA resulted from changing proportions of mesophyll tissue rather than from changes in VLA in monocots.

There are conflicting reports on the relationship between *K*_leaf_ and VLA (Scoffoni *et al.*, 2011; Carins Murphy *et al.*, 2012; Flexas *et al.*, 2013*b*). In the present study, we found that *K*_leaf_ was not correlated with VLA, VLA_major_, or VLA_minor_. However, *K*_leaf_ significantly increased with an increasing proportion of minor vein length in the genus *Oryza* (Fig. 2). In monocots, the water in major veins, as in minor veins, exits into the surrounding tissue, instead of into minor veins. Minor veins have a large surface area for exchange of xylem water with the surrounding mesophyll, and a short distance through which water travels outside the xylem (Sack and Holbrook, 2006). These results suggest that *K*_leaf_ in the genus *Oryza* may be driven by the cross-sectional conductivity of veins and outside xylem conductance (McKown *et al.*, 2010; Sommerville *et al.*, 2012).

After leaving the xylem, water must pass through liquid and gas phases before it reaches the sub-stomatal cavities. The water must first move through the bundle sheath, which is made up of parenchymatous cells wrapped around the veins, to mesophyll cells, and then diffuse into the intercellular airspace; or directly diffuse to intercellular airspace. Finally, the water escapes into the atmosphere via stomatal pores. The distance travelled by the water within leaves has been quantified in several ways (Brodribb *et al.*, 2007; Noblin *et al.*, 2008; North *et al.*, 2013), such as by measuring *D*_m_ and IVD. Brodribb *et al.* (2007) reported that *K*_leaf_ had a strongly negative relationship with *D*_m_ (in monocots IVD = 0.5*D*_m_) across species with a wide range of habitats and leaf structures. Furthermore, the relationships between *K*_leaf_ and IVD depend on water travel pathways and the water vapour concentration gradient between the intercellular airspace and atmosphere. In fact, the water in leaves turns into water vapour at mesophyll cell walls exposed to intercellular air space (Sack and Holbrook, 2006). Thus, if the liquid water supplement in leaves is not a limiting factor, an increase in *K*_leaf_ may occur via an increase in the mesophyll cell wall area exposed to the intercellular airspace. Indeed, Nardini *et al.* (2012) reported that *K*_leaf_ was enhanced by an increase in mesophyll porosity (the fraction of leaf mesophyll volume occupied by intercellular air space) under high irradiance.

The value of mesophyll porosity is relatively stable within the genus *Oryza* (Giuliani *et al.*, 2013). In other words, the volume of intercellular air space per leaf area depends on the proportion of mesophyll tissue in leaves. In rice, it has been shown that the proportion of mesophyll tissue in leaves is related to IVD (Smillie *et al.*, 2012) and *T*_leaf_ (Sack *et al.*, 2003). Early studies hypothesized that *K*_leaf_ in thick leaves should decline with increasing pathway length outside the xylem. However, experimental results show that *K*_leaf_ correlates with *T*_leaf_ across species, and across sun and shade leaves within a given species (Sack *et al.*, 2003; Zhang and Cao, 2009). This is because thicker leaves have more parallel flow pathways outside the xylem. Here we demonstrated that increases in IVD and *T*_leaf_ benefit *K*_leaf_ in the genus *Oryza* (Fig. 3).

### Effects of leaf N status on *K*_leaf_

N significantly influences rice leaf anatomy, structure, and function (Lee *et al.*, 2011). In the present study, leaf N content per leaf area had a significant positive effect on *K*_leaf_ (Fig. 4). Increased IVD and *T*_leaf_ under high N supplementation (data not shown) facilitates water evaporation at the cell wall surface, and this response could be one of the reasons why *K*_leaf_ increased with increasing N content per leaf area in leaves. Moreover, water flux across bundle sheath and mesophyll cells travels through either apoplastic, or cell-to-cell pathways, or both (Sack and Holbrook, 2006). In the cell-to-cell pathway, water molecules diffuse either across the plasma membrane or through plasmodesmata. Water channels, plasma membrane-intrinsic aquaporins (PIPs), play an important role in this process (Maggio and Joly, 1995; Pou *et al.*, 2013). Several studies have shown that PIP expression varies with N supply (Clarkson *et al.*, 2000; Guo *et al.*, 2007). It is reasonable to speculate that in rice PIPs are regulated by leaf N content per leaf area.

### Relationship between *K*_leaf_ and *g*_m_

Inside leaves, *K*_*leaf*_ and *g*_m_ are two traits which play central roles in determining gas exchange and plant performance (Sack and Holbrook, 2006; Flexas *et al.*, 2013*b*). However, very few studies have focused on their coordination; rather, the two traits have been studied independently in the past two decades. Recently, by summarizing the published data, Flexas *et al.* (2013*b*) reported that *K*_leaf_ was correlated with *g*_m_. In the present study, we found coordination of *K*_leaf_ and *g*_m_ in the genus *Oryza*, which provides further evidence that water and CO_2_ diffusion in the leaf share common pathways (Table 6). Many studies have found that *g*_m_ correlates with certain leaf structural traits in some species, particularly with the mesophyll cell surface area exposed to intercellular airspace per leaf area (*S*_m_) (Flexas *et al.*, 2008; Evans *et al.*, 2009; Flexas *et al.*, 2012). This correlation occurs because increasing *S*_m_ provides more pathways in parallel for CO_2_ diffusion. In fact, the mesophyll surface exposed to the intercellular airspace is the site at which water changes from liquid to vapour via evaporation. Cell wall thickness has been recognized as another important limiting factor for CO_2_ diffusion in the leaf. Interestingly, thick mesophyll cell walls may increase the extra-xylem apoplast path length, thereby increase *K*_leaf_. Further, membrane PIPs are known to facilitate transmembrane water transport as well as CO_2_ transport. For instance, Otto *et al.* (2010) reported a trade-off between water and CO_2_ permeability through membranes, depending on the proportion of PIP1 and PIP2 present.

Similar responses of *g*_m_ and *K*_leaf_ to various environmental factors, including temperature, light, leaf N status (Fig. 4; Supplementary Figure S4), and leaf water status, provide another line of evidence for their relationships (Flexas *et al.*, 2013*b*). However, the relative effects of leaf structural traits on *g*_m_ and *K*_leaf_, and the coordinated dynamics of *g*_m_ and *K*_leaf_ under various environmental conditions, needs to be clarified in the future.

In conclusion, there were significantly positive relationships between *K*_leaf_ and LMA, leaf area, proportion of minor vein length, IVD, *T*_leaf_, and leaf N content per leaf area in the genus *Oryza*, but *K*_leaf_ was independent of VLA. High *K*_leaf_ was associated with high *A*, *g*_s_, and *g*_m_. Our results indicate that leaf morpho-anatomical traits and leaf N content per leaf area had significant effects on *K*_leaf_, and suggest that more detailed anatomical and structural studies are needed to elucidate the impacts of leaf feature traits on *K*_leaf_ and gas exchange in grasses.

## Supplementary material

Supplementary data can be found at *JXB* online.

Supplementary Figure S1. Relationship between *αβ* values obtained using Yin’s method (Yin *et al.*, 2009) and the *Φ*_PSII_ and *Φ*_CO2_ slope method; and between *g*_m_ values estimated from a combination method with gas-exchange and Chl fluorescence, and the *A-Ci* curve-fitting method (b).

Supplementary Figure S2. Relationship between *K*_leaf_ and *A*, *g*_t_, *g*_s_, and *g*_m_.

Supplementary Figure S3. Relationship betweenVLA and LMA in the genus *Oryza*.

Supplementary Figure S4. Relationship between leaf N concentration and both *g*_s_ and *g*_m_.

## Funding

This work was supported by the Programme for Changjiang Scholars and Innovative Research Team in the University of China (IRT1247), Special Fund for Agro-scientific Research in the Public Interest of China from the Ministry of Agriculture (No. 201203096), and Fundamental Research Funds for the Central Universities (2012SC13).

## Supplementary Material

Supplementary Data
