# Supplementary material for: Leaf hydraulic conductance is coordinated with leaf morpho-anatomical traits and nitrogen status in the genus Oryza
Source: J Exp Bot. 2014 Nov 26;66(3):741–8. doi: 10.1093/jxb/eru434 (PMC4321541; doi:10.1093/jxb/eru434)
Supplement: Supplementary Data [file supp_66_3_741__index.html]

Leaf hydraulic conductance is coordinated with leaf morpho-anatomical traits and nitrogen status in the genus Oryza — Leaf hydraulic conductance is coordinated with leaf morpho-anatomical traits and nitrogen status in the genus Oryza — Supplementary Data 

# Leaf hydraulic conductance is coordinated with leaf morpho-anatomical traits and nitrogen status in the genus *Oryza*

## Supplementary Data

Data files

**Files in this Data Supplement:**

- Supplementary Data - Supplementary Data
