# Supplementary material for: Leaf hydraulic conductance is coordinated with leaf morpho-anatomical traits and nitrogen status in the genus Oryza
Source: J Exp Bot. 2014 Nov 26;66(3):741–8. doi: 10.1093/jxb/eru434 (PMC4321541; doi:10.1093/jxb/eru434)
Supplement: Supplementary Data [file supp_eru434_jexbot123315_file001.pdf]

Leaf hydraulic is conductance coordinated with leaf morpho-anatomic traits and nitrogen status in the genus *Oryza*

Dongliang Xiong, Tingting Yu, Tong Zhang, Yong Li, Shaobing Peng and Jianliang Huang*

Supplementary data

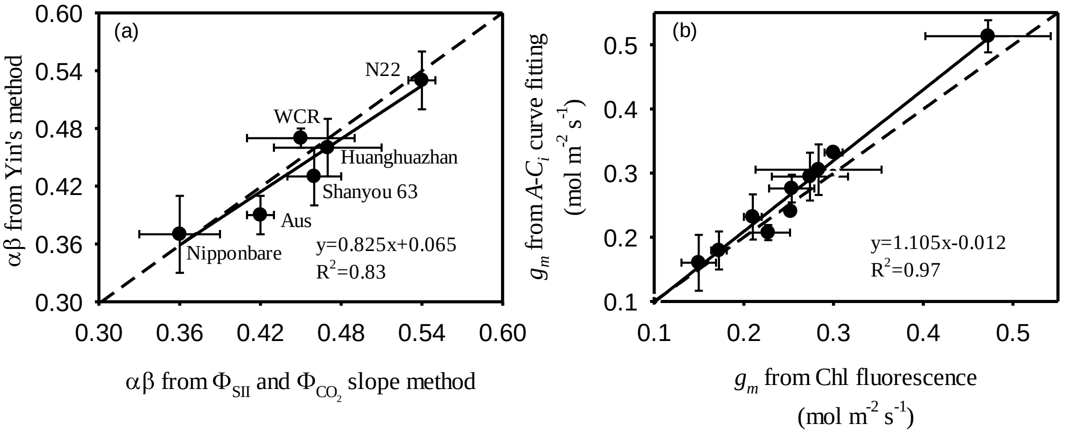

Figure S1 (a) Relationship between αβ obtained from Yin's method (Yin et al., 2009) and from the Φ_{PSII} and Φ_{CO_2} slope method. (b) Relationship between g_m estimated from two independent methods: combination method with gas-exchange and Chl fluorescence and *A-Ci* curve-fitting method (Sharkey et al., 2007).

References

Sharkey T.D., Bernacchi C.J., Farquhar G.D., Singsaas E.L., Fitting photosynthetic carbon dioxide response curves for C3 leaves, Plant, Cell and Environment, 30 (2007) 1035-1040.

Yin X, Struik PC, Romero P, Harbinson J, Evers JB, PE VDP, Vos J. 2009. Using combined measurements of gas exchange and chlorophyll fluorescence to estimate parameters of a biochemical C3 photosynthesis model: a critical appraisal and a new integrated approach applied to leaves in a wheat (*Triticum aestivum*) canopy. Plant, Cell and Environment 32, 448-464.

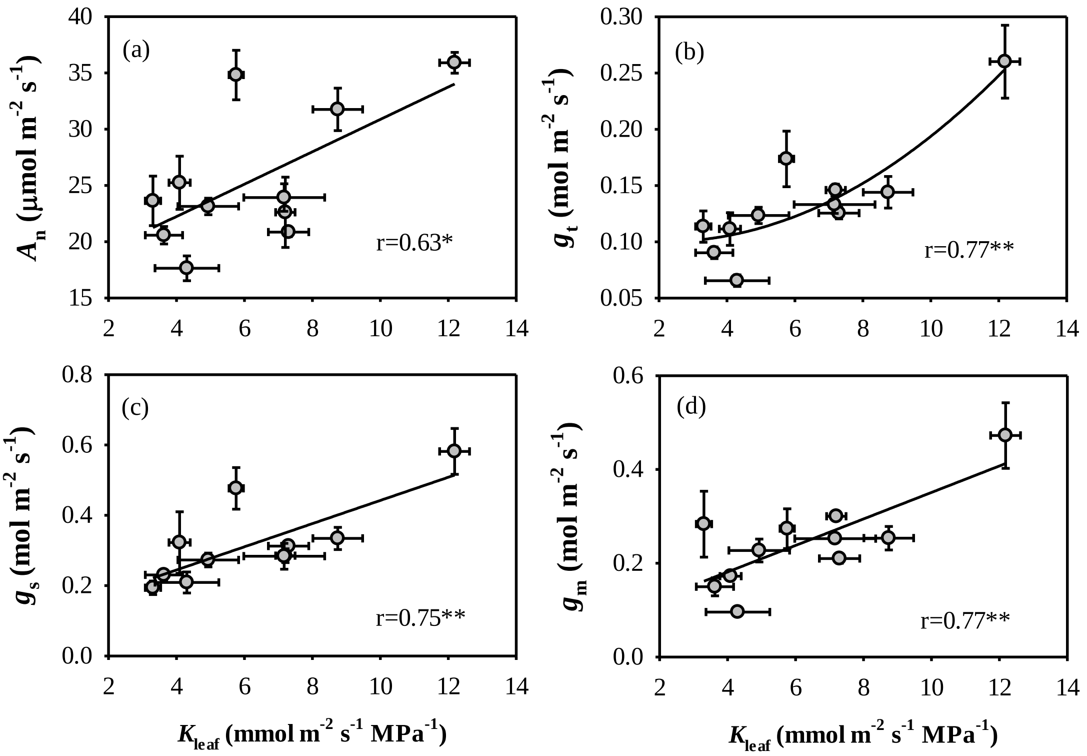

Figure S2 Relationships between leaf hydraulic conductance (K_{leaf}) and (a) photosynthesis (A), (b) total diffusion conductance (g_t), (c) stomatal conductance (g_s), and (d) mesophyll conductance (g_m).

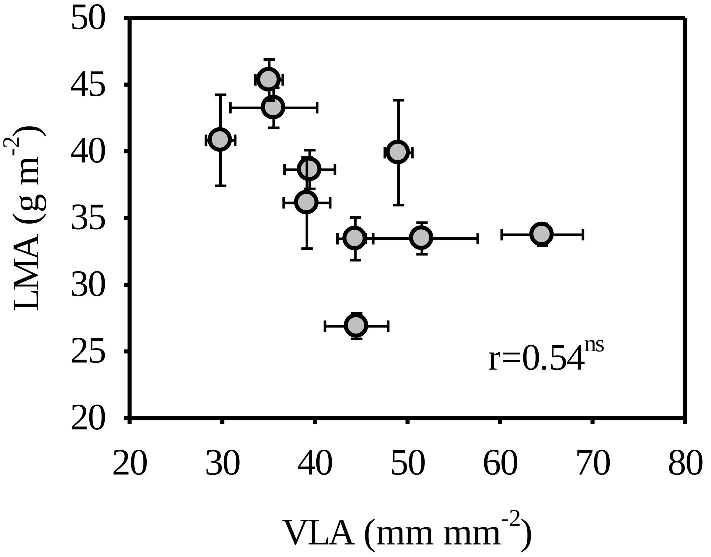

Figure S3 Relationship between leaf vein length per area (VLA) and leaf mass per area (LMA) in the genus *Oryza*.

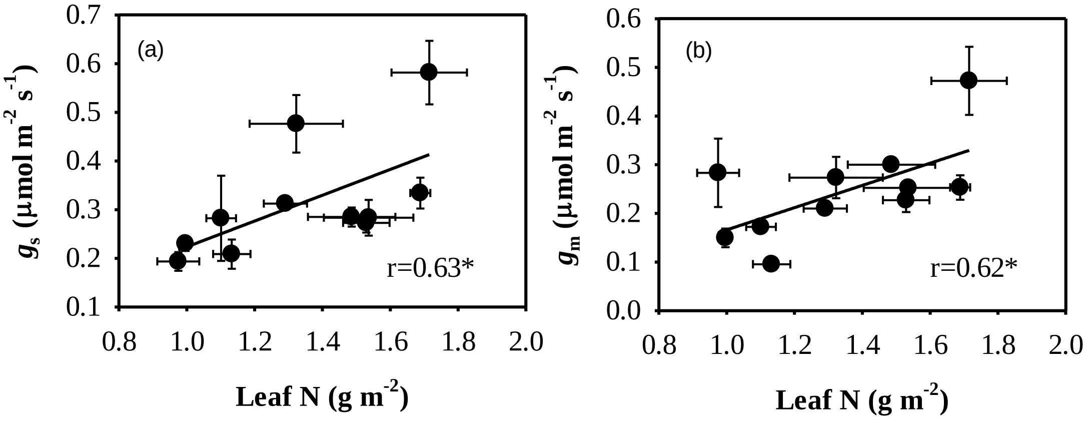

Figure S4 Relationships between leaf N concentration and (a) stomatal conductance (g_s), and (b) mesophyll conductance (g_m).
